# Supplementary material for: Saccharomyces boulardii (CNCM I-745) improves intestinal damage in sepsis by remodeling intestinal flora structure
Source: Front Cell Infect Microbiol. 2025 Jul 31;15:1602792. doi: 10.3389/fcimb.2025.1602792 (PMC12350329; doi:10.3389/fcimb.2025.1602792)
Supplement: Supplementary file 1 [file DataSheet1.zip › summary/summary/6_Taxonomy_community/1_Taxonomy_profiling/5_Family/Group/Family_abund_top30.Group.stacked_bar.pdf]

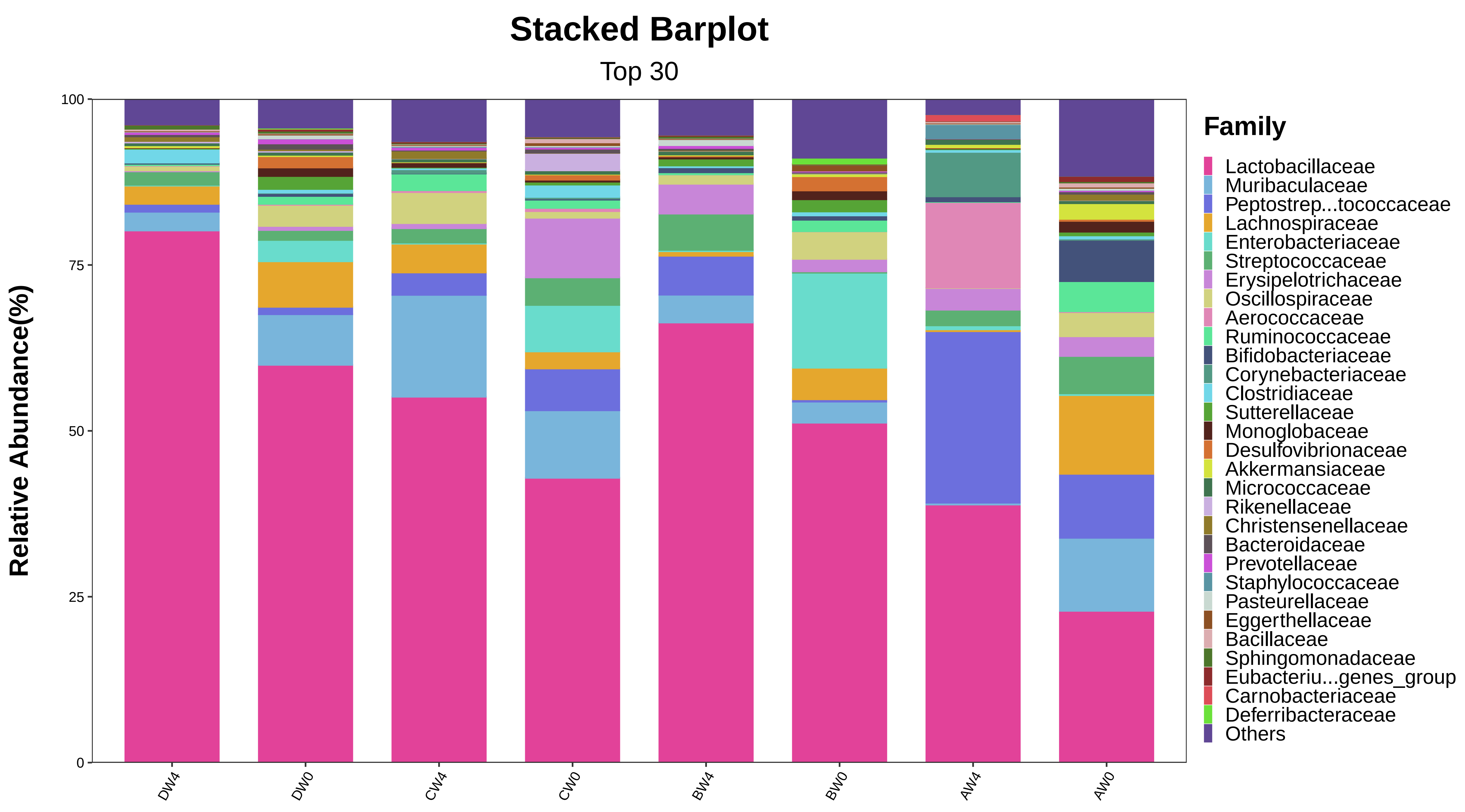
Stacked Barplot
Top 30
Relative Abundance(%)
100
75
50
25
0
DW4
DW0
CW4
CW0
BW4
BW0
AW4
AW0
Family
Lactobacillaceae
Muribaculaceae
Peptostrep...tococcaceae
Lachnospiraceae
Enterobacteriaceae
Streptococcaceae
Erysipelotrichaceae
Oscillospiraceae
Aerococcaceae
Ruminococcaceae
Bifidobacteriaceae
Corynebacteriaceae
Clostridiaceae
Sutterellaceae
Monoglobaceae
Desulfovibrionaceae
Akkermansiaceae
Micrococcaceae
Rikenellaceae
Christensenellaceae
Bacteroidaceae
Prevotellaceae
Staphylococcaceae
Pasteurellaceae
Eggerthellaceae
Bacillaceae
Sphingomonadaceae
Eubacteriu...genes_group
Carnobacteriaceae
Deferribacteraceae
Others
